# Flow Characterization in a Partially Liquefied Vitreous Humor

**DOI:** 10.1007/s11242-023-02052-x

**Published:** 2024-01-22

**Authors:** Anahid Khoobyar, Anita Penkova, Mark S. Humayun, Andrei Irimia, Satwindar Singh Sadhal

**Affiliations:** 1 Aerospace and Mechanical Engineering, USC Viterbi School of Engineering, Los Angeles, CA 90089, USA; 2 Saban Research Institute, Children’s Hospital Los Angeles, Los Angeles, CA 90027, USA; 3 Department of Ophthalmology, USC Keck School of Medicine, Los Angeles, CA 90033, USA; 4 USC Leonard Davis School of Gerontology, Los Angeles, CA 90089, USA; 5 Department of Biomedical Engineering, USC Viterbi School of Engineering, Los Angeles, CA 90089, USA; 6 USC Ginsburg Institute for Biomedical Therapeutics, Los Angeles, CA 90033, USA; 7 USC Roski Eye Institute, Los Angeles, CA 90033, USA

**Keywords:** Brinkman–Stokes, Darcy’s law, Liquefied vitreous humor

## Abstract

The purpose of this study is to systematically examine the basic fluid dynamics associated with a fully liquid region within a porous material. This work has come about as a result of our investigation on the ocular fluid dynamics and transport process in a partially liquefied vitreous humor. The liquid is modeled as a sphere with Stokes flow while the surrounding infinite porous region is described by Brinkman flow. The development here provides basic three-dimensional axisymmetric results on flow characterization and also serves to evaluate the limits of validity of Darcy flow analysis for the same geometry. In the Darcy flow model, the liquid region is also treated as a porous region with a much higher permeability. Therefore, both liquid and porous regions are modeled by Darcy’s law. Besides the analytical results from Brinkman–Stokes model, the simpler case of Darcy–Darcy flow for the same geometry has been provided. The results of both cases are compared and the differences between the two sets of results provide the range of validity of our computational model (Khoobyar et al. in J Heat Transf 144:031208, 2022). Some interesting fluid-dynamical aspects of the system are observed through the analysis. For the Darcy–Darcy system, the liquid region velocity is uniform throughout, as expected for potential flow. With the Brinkman–Stokes model, the liquid region has a paraboloidal profile with the maximum possible peak value of six times the far-field velocity in the porous medium. With the liquid region having a lower resistance, the flow tends to converge there for both models as it seeks the path of least resistance. As for the validation of the Darcy–Darcy model, it is a good approximation as far as the exterior flow is concerned. However, the liquid region flow profiles for the two models are different as noted. The current Brinkman–Stokes model has led to explicit analytical solutions for the flow field for both regions. This has permitted an asymptotic analysis giving deeper insight into the flow characterization.

## 1 Introduction

**Fig. 1:**
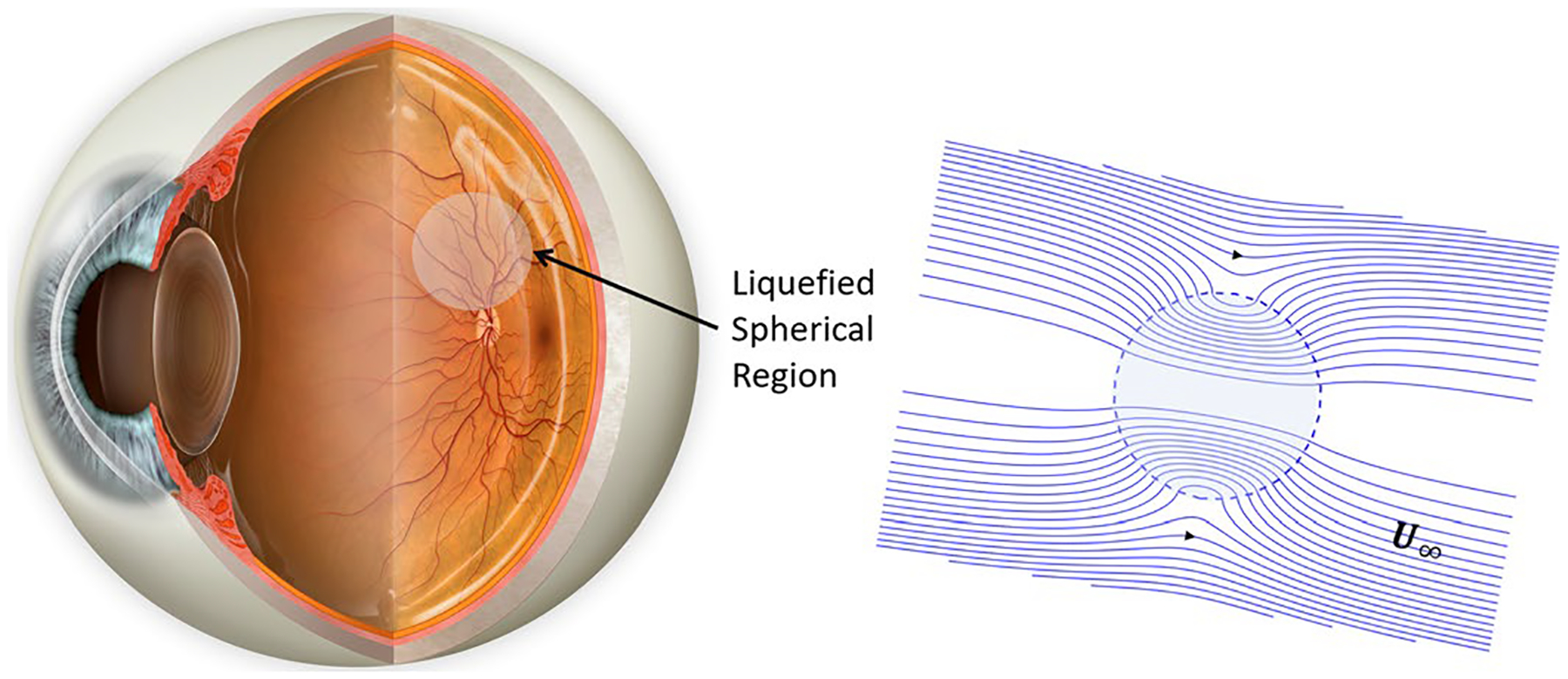
Liquefied spherical region in the vitreous humor. The localized flow around the spherical region is depicted on the right with uniform flow approximation in the vicinity

The motivation for this work has come about through our study of the fluid dynamics associated with a partially liquefied vitreous humor in connection with ocular drug delivery. The vitreous humor is gel-like transparent porous region in the eye between the lens and the retina. A succinct description of the structure of the vitreous has been provided by Penkova et al. (2020), as well as Nickerson et al. (2008), Sharif-Kashani and Hubschman (2011) and Silva et al. (2017). While the vitreous in a young person is a porous gel, with aging this develops liquid pockets (Bishop 2000) that affect the transport rates within the eye. Much of the earlier work on the transport process, the vitreous has been based on fully gel-like porous media without liquefaction (Xu et al. 2000; Ohtori and Tojo 1994; Haghjou et al. 2011; Stay et al. 2003; Balachandran and Barocas 2008; Balachandran 2010; Kathawate and Acharya 2008; Luo et al. 2022). In Fig. 1, a liquefied region is shown inside of the eye in vitreous humor. The region is assumed to be spherical and the flow around it in the porous region is also depicted. The liquefied region and as a fundamental problem could have any shape and it might be at numerous locations. For simplicity, only one spherical liquefied region is considered here.

It is only recently that the effect of partial liquefaction has been investigated. In a recent computational study (Khoobyar et al. 2022), we carried out the fluid-dynamical and mass-transfer analysis of the vitreous with a spherical liquefied region (whole sphere or segment) within the vitreous humor. The analysis was carried out using Darcy flow equations in the porous region as well as the liquid region. However, in the latter, the Darcy coefficient was taken to be much higher than the porous medium, effectively characterizing it as a liquid (we refer to it as the Darcy–Darcy system). This model makes the computation easier but does not allow us to satisfy the tangential velocity and shear stress continuity conditions at the liquid-gel interface since this is a reduced-order system where the viscous stress term $\mu \nabla^{2}\text{u}$ is replaced by a space-averaged quantity proportional to the locally averaged velocity, −(μ/K)u. The current study also provides the opportunity to examine the validity of the Darcy–Darcy approximation by considering Brinkman flow in the gel region and Stokes flow in the liquid. With the streamfunction formulation in each region being fourth order, the continuity of the tangential velocity and the shear stress continuity can be effectively carried out.

**Fig. 2:**
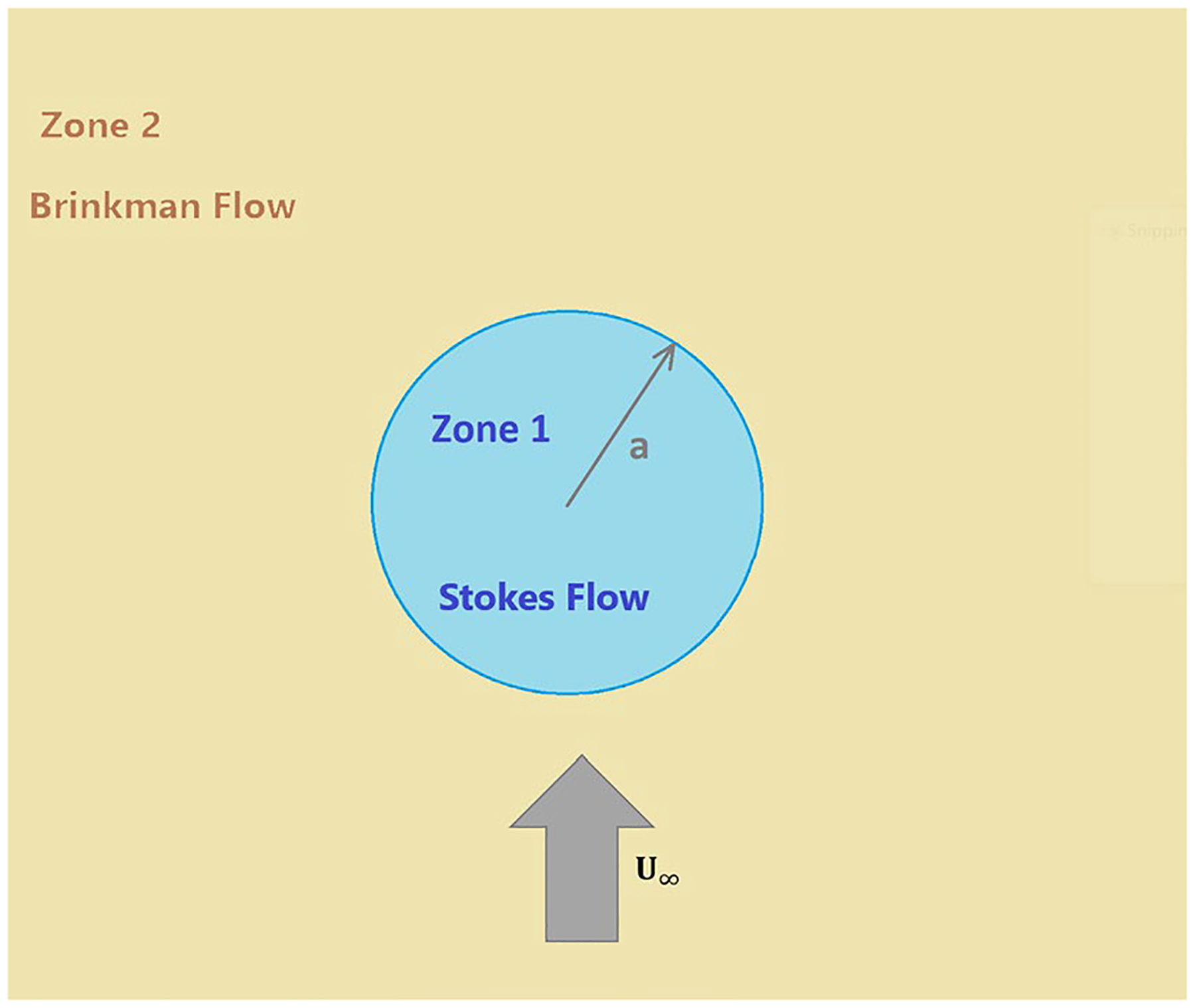
A liquefied sphere in an infinite porous medium

The system is approximated as a fundamental case that consists of a infinitely large porous medium (zone 2) enclosing a spherical liquid region of radius a (zone 1) as shown in Fig. 2. It is important to note that this study investigates a three-dimensional axisymmetric model. In general, a spherical liquid enclosure would encounter oncoming three-dimensional axisymmetric flow. However, as a first approximation, for a small spherical region, the oncoming flow can be considered to be uniform in the surrounding neighborhood of that region. Thus, the far-field velocity is taken to be uniform at $U_{\infty }$. A number of studies for this type of models have been conducted, such as Barman (1996), Deo and Gupta (2010), Grosan et al. (2010), Divoux et al. (2015), and Jaiswal and Gupta (2015). In this regard, the work of Grosan et al. (2010) seems to be most comprehensive since the authors have considered both regions to be Brinkman flow types with different Darcy coefficients. In addition, fluid penetration across the interface is accommodated with normal velocity continuity. However, due to the enormous complexity, the constant coefficients of the solution could not be explicitly expressed and appeared as a set of unsolved algebraic equations. For the current model, we have been able to obtain explicit expressions of velocity field in each region. These explicit expressions have facilitated an asymptotic analysis of some flow characteristics providing valuable insight into the fundamentals of the system. This could not be easily derived from the solution of Grosan et al. (2010) as a limiting case of interior Brinkman flow collapsing to Stokes flow.

## 2 Governing Equations

As mentioned in the Introduction, the porous region is treated as Brinkman flow while the enclosed liquid region is based on Stokes flow. Besides the Brinkman–Stokes results, we have additionally carried out the Darcy–Darcy calculation wherein the both regions are treated as Darcy flow with the interior being much more porous than the outer region. The results indicate considerable similarity between the Darcy–Darcy system and the Stokes-Brinkman system. However, some differences, such as the velocity distribution across the liquid sphere, are evident.

### 2.1 Steady Momentum and Mass Conservation Equations

As shown in Fig. 2, zone 1 is a liquefied spherical region governed by Stokes equation and zone 2 is an infinite porous medium surrounding the sphere and modeled by Brinkman equations. Subscripts 1 and 2 refer to liquid and porous regions, respectively. The continuity and momentum equation are

$$\text{Continuity:}\;\nabla \cdot {\bar{\text{V}}}_{i}=0,\;i=1,2,$$

$$\text{Stokes:}\;\mu \nabla^{2}{\bar{\text{V}}}_{1}=\nabla {\bar{P}}_{1},$$

$$\text{Brinkman:}\;\nabla {\bar{P}}_{2}=-\mu \frac{{\bar{\text{V}}}_{2}}{K_{2}}+\tilde{\mu }\nabla^{2}{\bar{\text{V}}}_{2},$$

where ${\bar{\text{V}}}_{i}$ is the velocity, ${\bar{P}}_{1}$ is the pressure, $K_{2}$ is the Darcy coefficient in the porous region, and μ is the dynamic viscosity of the liquid. In addition, $\tilde{\mu }$ represents the so-called Brinkman viscosity which has been the subject of much discussion (Koplik et al. 1983; Kolodziej 1988; Martys et al. 1994; Givler and Altobelli 1994; James and Davis 2001; Shavit et al. 2002; Valdes-Parada et al. 2007; Zaripov et al. 2019). The value of the viscosity ratio $\phi =\tilde{\mu }/\mu$ seems to have remained unsettled, especially for fibrous porous media, and therefore, we will use a range of values. The bar over the variables represents dimensional parameters. The index i corresponds to i=1,2 unless specified otherwise. We non-dimensionalize the pressure, velocity and the radial coordinate $\bar{r}$ as follows:

Dimensionless parameters:

(1) $$P_{1}=\frac{a{\bar{P}}_{1}}{\mu U_{\infty }},\;P_{2}=\frac{a{\bar{P}}_{2}}{\tilde{\mu }U_{\infty }},\;{\text{V}}_{i}=\frac{{\bar{\text{V}}}_{i}}{U_{\infty }},\;r=\frac{\bar{r}}{a},$$

where a is the radius of the spherical inclusion and $U_{\infty }$ is the far-field velocity. The latter is taken to be constant as a fundamental condition representing the idealized approximation of the flow in the vicinity of the spherical region. This is a standard far-field condition adopted by many investigators (Grosan et al. 2010; Jaiswal and Gupta 2015; Pop and Cheng 1992; Pop and Ingham 1996; Wang 2009; Leont’ev 2014; Deo and Gupta 2010; Deo and Ansari 2019). The velocity ${\text{V}}_{i}$ has components ${\text{V}}_{i}=\left(u_{i}{\text{e}}_{r}+v_{i}{\text{e}}_{\theta }\right)$ in the spherical coordinate system. We use the three-dimensional axisymmetric streamfunction formulation in both regions,

(2) $$u_{i}=\frac{1}{r^{2}\;\text{sin}\;\theta }\frac{\partial \psi_{i}}{\partial \theta },\;v_{i}=-\frac{1}{r\;\text{sin}\;\theta }\frac{\partial \psi_{i}}{\partial r},$$

by which the continuity equation is satisfied. Also, it should be noted that the viscosities used to make the pressure non-dimensional in liquefied and porous regions are the corresponding parameters, μ and $\tilde{\mu }$, respectively.

#### 2.1.1 Boundary and Interface Conditions

With the constant velocity at the far-field, the dimensionless streamfunction satisfies the condition,

(3) $${\psi_{2}|}_{r\to \infty }=\frac{1}{2}r^{2}\;{\text{sin}}^{2}\;\theta .$$

Also, in the liquid region where we require:

(4) $${u_{1}|}_{r\to 0}<\infty .$$

At the interface r=1, we have continuity of normal and tangential velocities, i.e.,

(5) $${u_{1}|}_{r=1}={u_{2}|}_{r=1},$$

(6) $${v_{1}|}_{r=1}={v_{2}|}_{r=1}.$$

In addition we have continuity of normal and shear stresses, i.e.,

(7) $${{\bar{\tau }}_{r\theta 1}|}_{r=1}={{\bar{\tau }}_{r\theta 2}|}_{r=1},$$

(8) $${{\bar{\tau }}_{rr1}|}_{r=1}={{\bar{\tau }}_{rr2}|}_{r=1},$$

or in terms of the velocities,

(9) $$\frac{\mu U_{\infty }}{a}{\left[\frac{1}{r}\frac{\partial u_{1}}{\partial \theta }+\frac{\partial v_{1}}{\partial r}-\frac{v_{1}}{r}\right]}_{r=1}=\frac{\tilde{\mu }U_{\infty }}{a}{\left[\frac{1}{r}\frac{\partial u_{2}}{\partial \theta }+\frac{\partial v_{2}}{\partial r}-\frac{v_{2}}{r}\right]}_{r=1},$$

(10) $$\frac{\mu U_{\infty }}{a}{\left[-P_{1}+2\frac{\partial u_{1}}{\partial r}\right]}_{r=1}=\frac{\tilde{\mu }U_{\infty }}{a}{\left[-P_{2}+2\frac{\partial u_{2}}{\partial r}\right]}_{r=1}.$$

For normal stress balance (Eqs. 8 and 10), we do not consider interfacial tension because with the spherical interface assumption, this effect may be ignored. Including the curvature effect will only add a constant pressure-difference term for the spherical interface. This is evident for the Stokes flow model (Taylor and Acrivos 1964) for low Reynolds number to the leading order.

The momentum equation in the two zones is given in spherical coordinates as follows:

#### 2.1.2 Interior Liquid Region


(11) $$\frac{\partial P_{1}}{\partial r}=\frac{1}{r^{2}}\frac{\partial }{\partial r}\left(r^{2}\frac{\partial u_{1}}{\partial r}\right)+\frac{1}{r^{2}\;\text{sin}\;\theta }\frac{\partial }{\partial \theta }\left(\text{sin}\;\theta \frac{\partial u_{1}}{\partial \theta }\right)-\frac{2u_{1}}{r^{2}}-\frac{2}{r^{2}}\frac{\partial v_{1}}{\partial \theta }-\frac{2v_{1}\;\text{cot}\;\theta }{r^{2}},$$

(12) $$\frac{1}{r}\frac{\partial P_{1}}{\partial \theta }=\frac{1}{r^{2}}\frac{\partial }{\partial r}\left(r^{2}\frac{\partial v_{1}}{\partial r}\right)+\frac{1}{r^{2}\;\text{sin}\;\theta }\frac{\partial }{\partial \theta }\left(\text{sin}\;\theta \frac{\partial v_{1}}{\partial \theta }\right)+\frac{2}{r^{2}}\frac{\partial u_{1}}{\partial \theta }-\frac{v_{1}}{r^{2}\;{\text{sin}}^{2}\;\theta }$$

In terms of the streamfunction, we have (Happel and Brenner 1983)

(13) $$D^{4}\psi_{1}=0,$$

where $D^{2}$ is the Stokes operator

(14) $$D^{2}=\frac{\partial^{2}}{\partial r^{2}}+\frac{\text{sin}\;\theta }{r^{2}}\frac{\partial }{\partial \theta }\left(\frac{1}{\text{sin}\;\theta }\frac{\partial }{\partial \theta }\right).$$

For the detailed derivation of the pressure and streamfunction for the interior liquid region, please refer to the Appendix (A.1.1).

#### 2.1.3 Exterior Porous Region

The analytical derivation for Brinkman flow across a spherical region has been carried out in many studies before (Pop and Cheng 1992; Pop and Ingham 1996; Wang 2009; Leont’ev 2014). We follow these studies but attempt to present fully analytical explicit results.

By defining the dimensionless parameter, $\alpha =a/\sqrt{K_{2}}$ (see Grosan et al. (2010)), we have the following for the momentum equation in the porous region:

(15) $$\frac{\partial P_{2}}{\partial r}=\;-\alpha^{2}\frac{\mu }{\tilde{\mu }}u_{2}+[\frac{\partial^{2}u_{2}}{\partial r^{2}}+\frac{2}{r}\frac{\partial u_{2}}{\partial r}+\frac{1}{r^{2}}\frac{\partial^{2}u_{2}}{\partial \theta^{2}}+\frac{\text{cot}\;\theta }{r^{2}}\frac{\partial u_{2}}{\partial \theta }-\frac{2u_{2}}{r^{2}}-\frac{2}{r^{2}}\frac{\partial v_{2}}{\partial \theta }-\frac{2v_{2}\;\text{cot}\;\theta }{r^{2}}],$$

(16) $$\frac{1}{r}\frac{\partial P_{2}}{\partial \theta }=\;-\alpha^{2}\frac{\mu }{\tilde{\mu }}v_{2}+[\frac{\partial^{2}v_{2}}{\partial r^{2}}+\frac{2}{r}\frac{\partial v_{2}}{\partial r}+\frac{1}{r^{2}}\frac{\partial^{2}v_{2}}{\partial \theta^{2}}+\frac{\text{cot}\;\theta }{r^{2}}\frac{\partial v_{2}}{\partial \theta }+\frac{2}{r^{2}}\frac{\partial u_{2}}{\partial \theta }-\frac{v_{2}}{r^{2}\;{\text{sin}}^{2}\;\theta }],$$

which, in terms of the streamfunction, may be expressed as (Grosan et al. 2010; Jaiswal and Gupta 2015; Deo and Gupta 2010; Barman 1996)

(17) $$D^{2}\left[D^{2}-\alpha^{2}\right]\psi_{2}=0,$$

where $D^{2}$ is defined in Eq. (14).

We have expanded more on the next steps of obtaining the pressure and streamfunction in the porous region in the Appendix, section A.1.2.

### 2.2 Final Expressions for the Streamfunctions

The final, explicit expressions of pressure and streamfunction for both regions are as follows:

#### 2.2.1 Porous Region


(18) $$P_{2}=\left[-\frac{\alpha^{2}r}{\phi }+\frac{\left(\frac{1}{6}\frac{1}{\phi +\frac{3}{2}}\frac{\alpha^{2}}{r^{2}}+\frac{1}{\phi r^{2}}\right)\left(1+\frac{1}{\alpha }\right)+\frac{\alpha }{3\phi r^{2}}+e^{-\alpha \left(r-1\right)}\left(1-\frac{1}{\phi }\right)\left(-\alpha +\frac{1}{r}+\frac{1}{\alpha r^{2}}\right)}{\left(\frac{\phi }{6\left(\phi +\frac{3}{2}\right)}+\frac{3\phi }{\alpha^{2}}\right)\left(1+\frac{1}{\alpha }\right)+\left(\phi -\frac{2}{3}\right)\frac{1}{\alpha }}\right]\text{cos}\;\theta ,$$

and

(19) $$\psi_{2}=\left[\frac{1}{2}r^{2}+\frac{\left(\frac{1}{6r}\frac{\phi }{\phi +\frac{3}{2}}+\frac{1}{\alpha^{2}r}\right)\left(1+\frac{1}{\alpha }\right)+\frac{1}{3\alpha r}-\frac{e^{-\alpha \left(r-1\right)}}{\alpha^{2}}\left(1+\frac{1}{\alpha r}\right)}{\left(\frac{\phi }{6\left(\phi +\frac{3}{2}\right)}+\frac{3\phi }{\alpha^{2}}\right)\left(1+\frac{1}{\alpha }\right)+\left(\phi -\frac{2}{3}\right)\frac{1}{\alpha }}\right]{\text{sin}}^{2}\;\theta .$$

#### 2.2.2 Liquefied Region


(20) $$P_{1}=\left[\frac{-\frac{5\phi }{\phi +\frac{3}{2}}\left(1+\frac{1}{\alpha }\right)r}{\left(\frac{\phi }{6\left(\phi +\frac{3}{2}\right)}+\frac{3\phi }{\alpha^{2}}\right)\left(1+\frac{1}{\alpha }\right)+\left(\phi -\frac{2}{3}\right)\frac{1}{\alpha }}\right]\text{cos}\;\theta ,$$

and

(21) $$\psi_{1}=\left[\frac{1}{2}r^{2}+\frac{\frac{\phi }{\phi +\frac{3}{2}}\left(\frac{5r^{2}}{12}-\frac{r^{4}}{4}\right)\left(1+\frac{1}{\alpha }\right)+\frac{r^{2}}{3\alpha }}{\left(\frac{\phi }{6\left(\phi +\frac{3}{2}\right)}+\frac{3\phi }{\alpha^{2}}\right)\left(1+\frac{1}{\alpha }\right)+\left(\phi -\frac{2}{3}\right)\frac{1}{\alpha }}\right]{\text{sin}}^{2}\;\theta .$$

## 3 Darcy–Darcy Solution

For the sake of completeness and comparative analysis, the Darcy flow analysis in both regions is also provided. Some previous works have studied the analytical model for a porous sphere embedded within another infinite porous medium (Deo and Gupta 2010; Deo and Ansari 2019). In these studies, both regions are governed with Brinkman models, while for the analytical model developed in this section, porous and liquid region are both governed by Darcy law but, as mentioned already, the permeability of the liquid region is assumed to be much higher than the porous region. This model allows the fluid in zone 1 to be much more freely flowing than zone 2. As before, the subscripts 1 and 2 refer to liquid and porous regions, respectively. The dimensional governing equations are as follows:

(22) $$\text{Continuity:}\;\nabla \cdot {\bar{\text{V}}}_{i}=0,\;\nabla^{2}{\bar{P}}_{i}=0,\;i=1,2,$$

(23) $$\text{Darcy}\;\text{law:}\;\nabla {\bar{P}}_{i}=-\frac{\mu }{K_{i}}{\bar{\text{V}}}_{i},$$

(24) $$\text{where},\;\frac{K_{1}}{K_{2}}\gg 1.$$

At the far field, in porous region pressure should be equal to

(25) $${P_{2}|}_{r\to \infty }=\frac{-a^{2}}{K_{2}}r\;\text{cos}\;\theta .$$

At the interface of the liquid and porous regions, we have pressure and normal velocities continuity at r=1,

(26) $$P_{1}=P_{2}\;\text{at}\;r=1,$$

(27) $${K_{1}\frac{\partial P_{1}}{\partial r}|}_{r=1}={K_{2}\frac{\partial P_{2}}{\partial r}|}_{r=1}.$$

We obtain the following expressions for the pressure (for more details, please refer to A.2):

(28) $$P_{1}=\frac{-3a^{2}}{2K_{2}+K_{1}}r\;\text{cos}\;\theta ,$$

(29) $$P_{2}=\left[\frac{-a^{2}}{K_{2}}r+\frac{a^{2}}{r^{2}}\left(\frac{1}{K_{2}}-\frac{3}{2K_{2}+K_{1}}\right)\right]\text{cos}\;\theta .$$

Using Equation (23), we obtain the velocities,

(30) $$u_{1}=\frac{3K_{1}a^{2}}{\mu \left(2K_{2}+K_{1}\right)}\text{cos}\;\theta ,$$

(31) $$v_{1}=\frac{-3K_{1}a^{2}}{\mu \left(2K_{2}+K_{1}\right)}\text{sin}\;\theta ,$$

(32) $$u_{2}=\left[\frac{K_{1}a^{2}}{\mu K_{2}}+\frac{2a^{2}}{\mu r^{3}}\left(\frac{K_{1}}{K_{2}}-\frac{3K_{1}}{2K_{2}+K_{1}}\right)\right]\text{cos}\;\theta ,$$

(33) $$v_{2}=\left[-\frac{K_{1}a^{2}}{\mu K_{2}}-\frac{a^{2}}{\mu r^{3}}\left(\frac{K_{1}}{K_{2}}-\frac{3K_{1}}{2K_{2}+K_{1}}\right)\right]\text{sin}\;\theta .$$

Next, using the streamfunction formulation in Eq. (2), it is not difficult to obtain

(34) $$\psi_{1}=\frac{3K_{1}}{2\left(2K_{2}+K_{1}\right)}r^{2}\;{\text{sin}}^{2}\;\theta ,$$

(35) $$\psi_{2}=\left[\frac{r^{2}}{2}+\frac{1}{r}\left(\frac{K_{1}-K_{2}}{2K_{2}+K_{1}}\right)\right]{\text{sin}}^{2}\;\theta .$$

## 4 Results

**Fig. 3:**
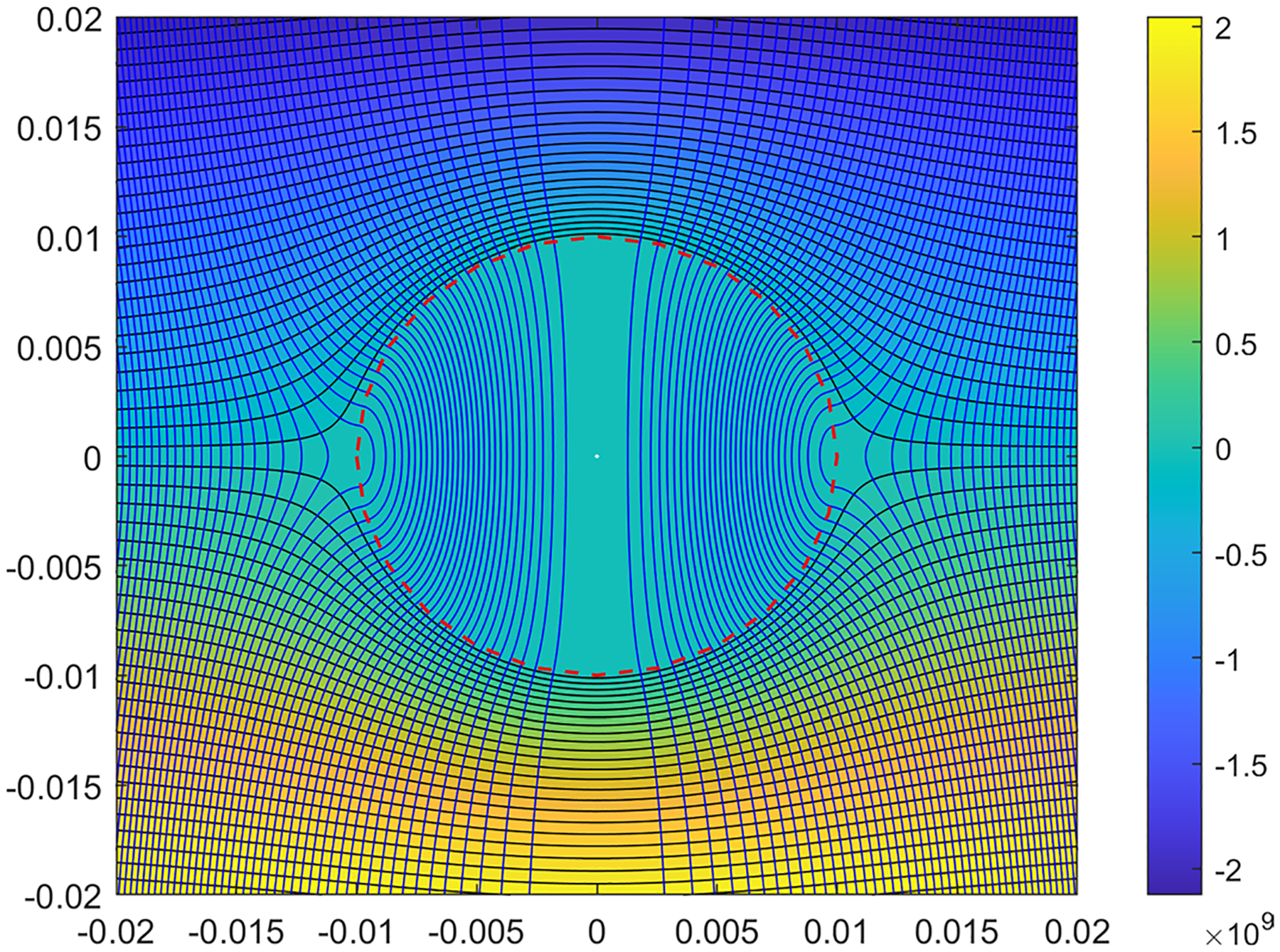
Streamlines and isobars of the Brinkman–Stokes model for $\alpha =1.12\times 10^{6}$, and ϕ=1.2. Black lines represent the streamlines. The liquefied region is separated from the porous region with a red dashed circle

**Fig. 4:**
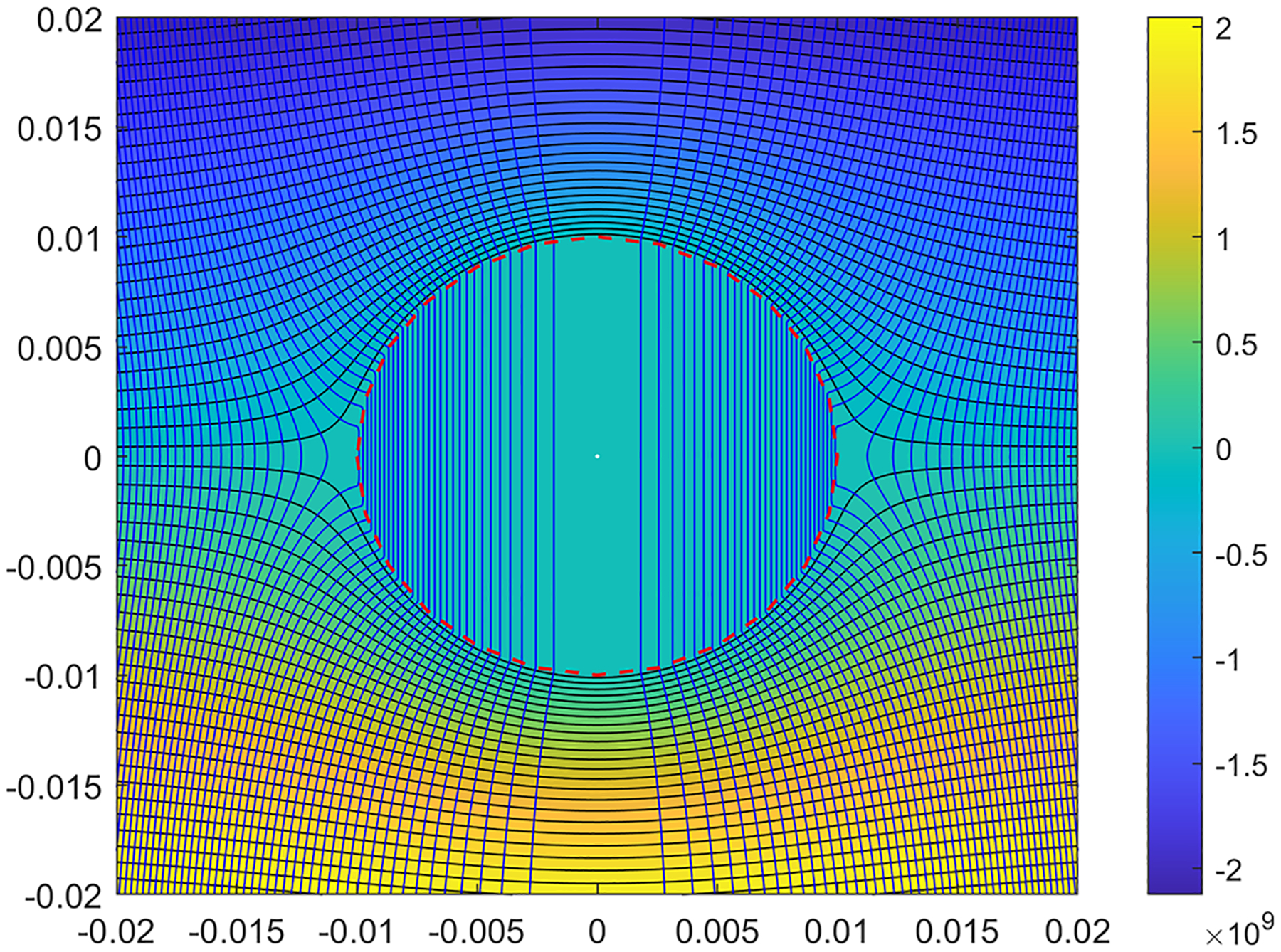
Streamlines and isobars for the Darcy–Darcy model

Using the fully analytical expressions for the three-dimensional axisymmetric stream-functions and pressure (Eqs. (18)–(21), (28)–(29) and (34)–(35)), the plots for these values have been constructed in a parametric form. These are expressed as isobars and streamlines which are orthogonal to each other. Figure 3 represents the set of results for the Stokes-Brinkman model and Fig. 4 shows the corresponding plots for the Darcy–Darcy model.

From these figures, one can observe that for the streamlines in the liquefied region in Darcy–Darcy analytical model absolutely no curvature exists, whereas in Brinkman–Stokes model as the streamlines enter the liquefied region slight curvature is seen. While we used several values of values of ϕ in the range 0.8≤ϕ≤2, we have found that the flow field is quite insensitive to the value and only one set of results is presented with ϕ=1.2. The straight streamlines for the Darcy–Darcy model are not unexpected since such behavior is typical for systems governed by potential theory.

### 4.1 Slip Velocity

Another difference in the two models is mismatch of the tangential velocities at the interface of the two regions. With the Brinkman–Stokes model, tangential velocities at interface are equal to each other, while for the Darcy–Darcy model, there is a jump in the tangential velocities at the interface. The velocity values are:

(36) $${v_{1}|}_{r=1}=\frac{-3K_{1}}{2K_{2}+K_{1}}\text{sin}\;\theta ,$$

(37) $$\begin{array}{c} {v_{2}|}_{r=1}=\frac{-3K_{2}}{2K_{2}+K_{1}}\text{sin}\;\theta , \end{array}$$

resulting in a slip velocity:

(38) $${\left(v_{1}-v_{2}\right)|}_{r=1}=-3\frac{K_{1}-K_{2}}{2K_{2}+K_{1}}\text{sin}\;\theta .$$

### 4.2 Flow Across the Liquid Region

Based on the flow across the liquid region, we attempt to establish the parametric range of validity of the Darcy–Darcy model. That is, what value of the K-ratio should be used so that the results resemble the Stokes-Brinkman results for the integrated flow across the liquid region. The dimensional flow across the liquid sphere for both analytical models is as follows:

(39) $$\text{Brinkman–Stokes}\;:\bar{Q}=2aU_{\infty }\left\{1+\frac{\frac{1}{3}\left[\frac{\phi }{\phi +\frac{3}{2}}\left(1+\frac{1}{\alpha }\right)-\frac{2}{\alpha }\right]}{\left(\frac{\phi }{6\left(\phi +\frac{3}{2}\right)}+\frac{3\phi }{\alpha^{2}}\right)\left(1+\frac{1}{\alpha }\right)+\left(\phi -\frac{2}{3}\right)\frac{1}{\alpha }}\right\}$$

(40) $$\text{Darcy}\;\text{Darcy}\;:\bar{Q}=2aU_{\infty }\left(\frac{3K_{1}}{2K_{2}+K_{1}}\right)$$

**Fig. 5:**
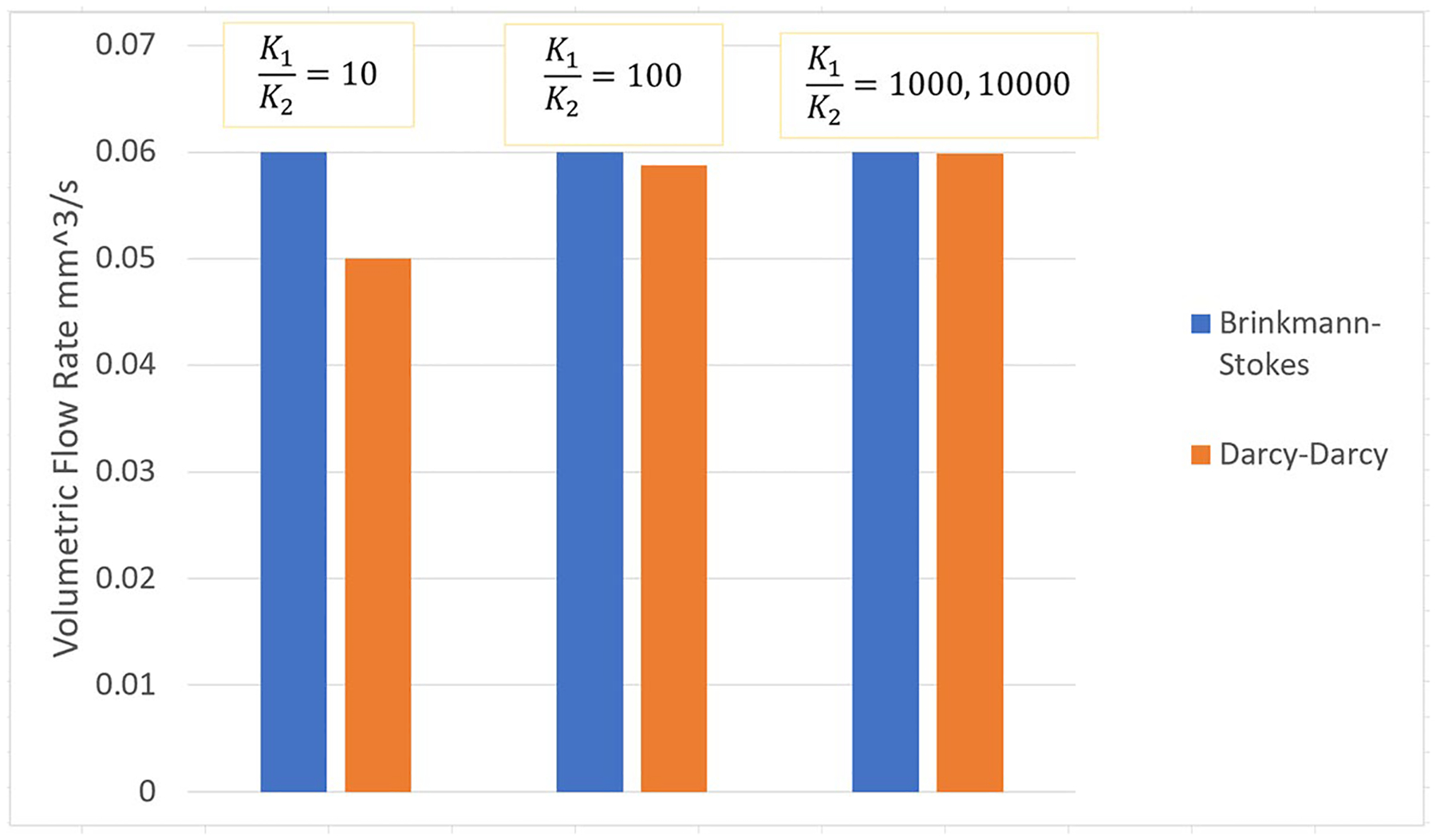
Changes of flow across the liquid region for Darcy–Darcy analytical model for varying K-ratio and how it compares with the Brinkman–Stokes case

Using a range values for the parameters, the rate of the volumetric flow entering the liquefied sphere have been calculated for both cases for varying K-ratios. In Fig. 5, the flow across the liquid sphere is derived for $a=0.01\;\text{m},\;U_{\infty }=1\;\text{m}/\text{s}$ and $K_{2}=9\times 10^{-12}\;{\text{m}}^{2}/\text{Pa.s}$ in both analytical models with varying K-ratios, and $\phi =\tilde{\mu }/\mu =0.8,1.0,1.1,1.2,1.3,1.5$ and 2.0 for the Brinkman–Stokes model. As mentioned earlier, the value of viscosity ratio ϕ is a subject of much discussion and we have therefore a range of values. The flow streamlines are shown in Figs. 3 and 4 where the calculations are based on ϕ=1.2.

### 4.3 Velocity Distribution

In this section, variation of the velocity over selected planes is calculated in terms of the radial distance from the polar axis. To begin with, the r and θ components of the velocity distribution in Brinkman–Stokes and Darcy–Darcy models are as follows:

**Brinkman–**Stokes:

For r>1:

(41) $$u_{2}=\left[1+\frac{\frac{1}{r^{3}}\left(1+\frac{1}{\alpha }\right)\left(\frac{\phi }{3\left(\phi +\frac{3}{2}\right)}+\frac{2}{\alpha^{2}}\right)+\frac{2}{3\alpha r^{3}}-\frac{2e^{-\alpha \left(r-1\right)}}{r^{2}\alpha^{3}}\left(1+\frac{1}{\alpha r}\right)}{\left(\frac{\phi }{6\left(\phi +\frac{3}{2}\right)}+\frac{3\phi }{\alpha^{2}}\right)\left(1+\frac{1}{\alpha }\right)+\left(\phi -\frac{2}{3}\right)\frac{1}{\alpha }}\right]\text{cos}\;\theta ,$$

(42) $$\begin{array}{c} v_{2}=\left[-1+\frac{\frac{1}{r^{3}}\left(1+\frac{1}{\alpha }\right)\left(\frac{\phi }{6\left(\phi +\frac{3}{2}\right)}+\frac{1}{\alpha^{2}}\right)+\frac{1}{3\alpha r^{3}}-\frac{e^{-\alpha \left(r-1\right)}}{r^{2}\alpha^{2}}\left(1+\frac{2}{\alpha r}\right)}{\left(\frac{\phi }{6\left(\phi +\frac{3}{2}\right)}+\frac{3\phi }{\alpha^{2}}\right)\left(1+\frac{1}{\alpha }\right)+\left(\phi -\frac{2}{3}\right)\frac{1}{\alpha }}\right]\text{sin}\;\theta . \end{array}$$

For 0≤r≤1:

(43) $$\begin{array}{c} u_{1}=\left[1+\frac{\frac{-\phi }{\phi +\frac{3}{2}}\left(\frac{r^{2}}{2}-\frac{5}{6}\right)\left(1+\frac{1}{\alpha }\right)+\frac{2}{3\alpha }}{\left(\frac{\phi }{6\left(\phi +\frac{3}{2}\right)}+\frac{3\phi }{\alpha^{2}}\right)\left(1+\frac{1}{\alpha }\right)+\left(\phi -\frac{2}{3}\right)\frac{1}{\alpha }}\right]\text{cos}\;\theta , \end{array}$$

(44) $$v_{1}=\left[-1+\frac{\frac{\phi }{\phi +\frac{3}{2}}\left(r^{2}-\frac{5}{6}\right)\left(1+\frac{1}{\alpha }\right)-\frac{2}{3\alpha }}{\left(\frac{\phi }{6\left(\phi +\frac{3}{2}\right)}+\frac{3\phi }{\alpha^{2}}\right)\left(1+\frac{1}{\alpha }\right)+\left(\phi -\frac{2}{3}\right)\frac{1}{\alpha }}\right]\text{sin}\;\theta .$$

**Darcy–**Darcy:

For r>1:

(45) $$u_{2}=\left[1+\frac{2}{r^{3}}\left(\frac{K_{1}-K_{2}}{2K_{2}+K_{1}}\right)\right]\text{cos}\;\theta ,$$

(46) $$\begin{array}{c} v_{2}=\left[-1+\frac{1}{r^{3}}\left(\frac{K_{1}-K_{2}}{2K_{2}+K_{1}}\right)\right]\text{sin}\;\theta . \end{array}$$

For 0≤r≤1:

(47) $$u_{1}=\left[\frac{3K_{1}}{2K_{2}+K_{1}}\right]\text{cos}\;\theta ,$$

(48) $$\begin{array}{c} v_{1}=\left[-\frac{3K_{1}}{2K_{2}+K_{1}}\right]\text{sin}\;\theta . \end{array}$$

**Fig. 6:**
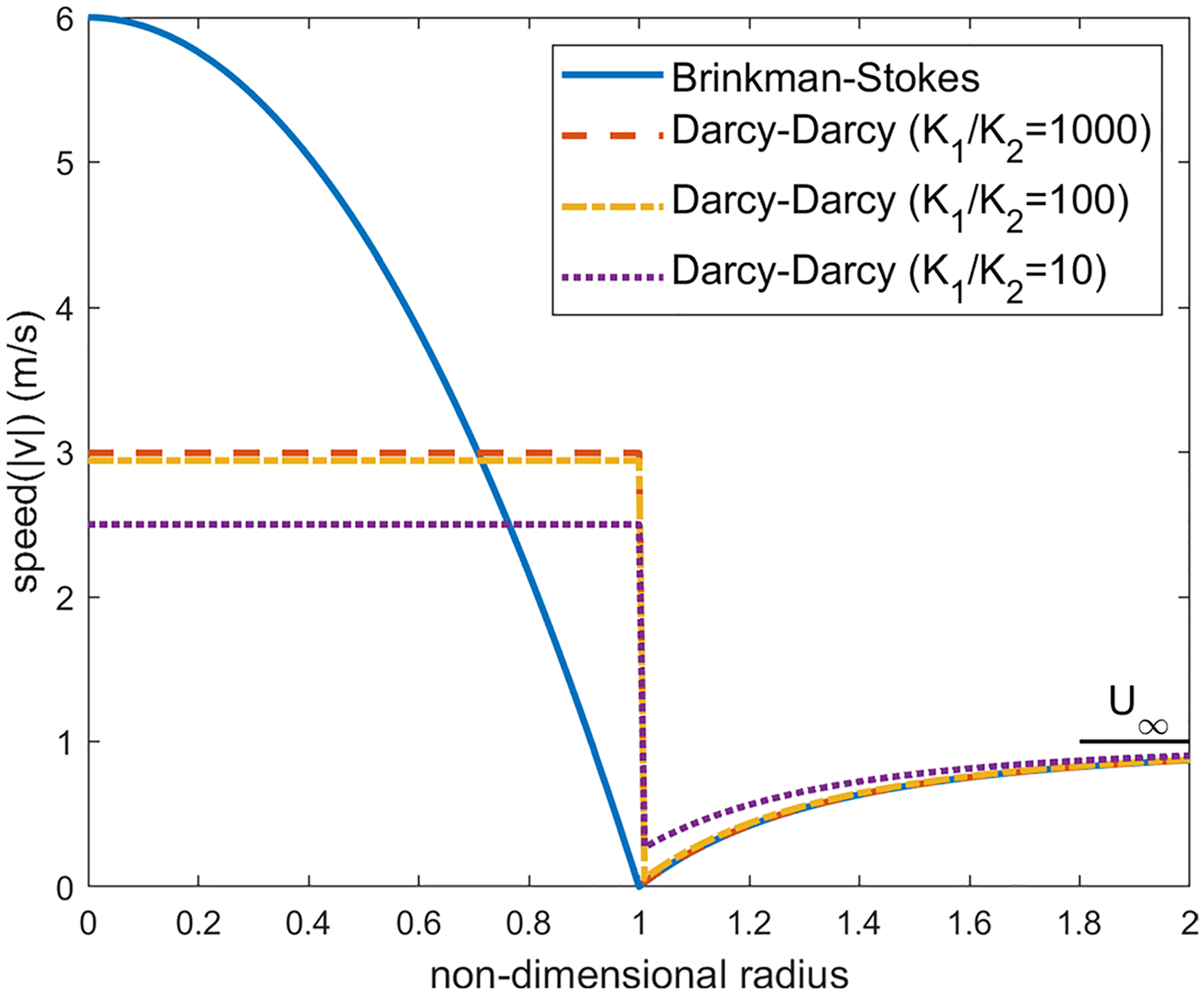
Distribution of the velocity of the equatorial plane for ϕ=1.2,a=1cm and $\alpha =1.12\times 10^{6}$

We first examine the flow across the equatorial plane corresponding to $\theta =\frac{\pi }{2}$. For the values $K_{1}/K_{2}=1000$, a=1cm and $\alpha =1.12\times 10^{6}$, the velocity distribution on the equatorial plane is plotted in Fig. 6. We have applied several values for the viscosity ratio ϕ in the range 0.8≤ϕ≤2.0 and obtained the almost same figure for each value. In both models, as one can observe, the velocity in the liquid region is much higher than the porous region. This is expected as the streamlines converge into the liquid sphere since it offers less resistance to the pathway of the flow compared with the porous region.

With the Darcy–Darcy model, the velocity remains uniform in the liquid region (this constant value is approximately the average of velocity in the liquid sphere in the Brinkman–Stokes model). As we move from the liquid region on the equatorial plane toward infinity, a jump occurs on the liquid-porous interface as the continuity of the velocity on the interface is not satisfied in this case. The porous region velocity in the Darcy–Darcy model increases steadily with r from the minimum at the interface up to the far-field value.

As we decrease the permeability ratio in the Darcy equation, the velocity on the equatorial plane in the porous region drifts away from the velocity distribution in the Brinkman–Stokes model as depicted in Fig. 6. The ratio $K_{1}/K_{2}=1000$ which was used in the finite element model in the study carried out by our group (Khoobyar et al. 2022) is a reasonable permeability ratio in this regard.

For the Brinkman–Stokes model, on the equatorial plane, the maximum velocity in the liquid region occurs at the center of the sphere. This is also discussed in Sect. 4.3.1. As we move from the center, the magnitude of the velocity drops when getting closer to the interface. The minimum value in the liquid region is reached at the interface. The large flow rate in the liquid region comes at the expense of lower velocity in the porous region in the vicinity of the spherical interface. On the equatorial plane, the velocity decreases from r=1, reaches a minimum value and then catches up to far-field value $U_{\infty }$. The minimum is not obvious in Fig. 6. Therefore, in order to fully understand the flow characterization, for the Brinkman–Stokes model, we have carried out an asymptotic analysis with α≫1 for the velocity in the equatorial plane in both the liquid and porous regions.

#### 4.3.1 Asymptotic Analysis on the Equatorial Plane

With the fully analytical explicit expression for the velocity fields (Eqs. 41–44), it is possible to carry out an asymptotic analysis to obtain more insight into the flow field. In this subsection, we examine two items of interest concerning the velocity in the equatorial plane for the Brinkman–Stokes model. The first is the peak velocity in the liquid region. As mentioned, this occurs at r=0, and we refer to it as the centerpoint velocity. The second is the location of the minimum velocity in the porous region.

##### Centerpoint Velocity

Using Eq. (43), the velocity on the equatorial plane at r=0 is given by

(49) $${u_{1}|}_{r,\theta \to 0}=\frac{\left(\frac{\phi }{\phi +\frac{3}{2}}+\frac{3\phi }{\alpha^{2}}\right)\left(1+\frac{1}{\alpha }\right)+\frac{\phi }{\alpha }}{\left(\frac{\phi }{6\left(\phi +\frac{3}{2}\right)}+\frac{3\phi }{\alpha^{2}}\right)\left(1+\frac{1}{\alpha }\right)+\left(\phi -\frac{2}{3}\right)\frac{1}{\alpha }},$$

Neglecting terms of $O\left(\frac{1}{\alpha^{2}}\right)$, we obtain

(50) $${u_{1}|}_{r,\theta \to 0}=\frac{\frac{\phi }{\left(\phi +\frac{3}{2}\right)}\left(1+\frac{1}{\alpha }\right)+\frac{\phi }{\alpha }}{\frac{\phi }{6\left(\phi +\frac{3}{2}\right)}\left(1+\frac{1}{\alpha }\right)+\left(\phi -\frac{2}{3}\right)\frac{1}{\alpha }}+O\left(\frac{1}{\alpha^{2}}\right),$$

which after some rearrangement, can be written as

(51) $${u_{1}|}_{r,\theta \to 0}=6\left[1+\frac{1}{\alpha }\left(\frac{5}{2}+\phi \right)\right]{\left[1+\frac{6}{\alpha \phi }\left(\phi^{2}+\phi -1\right)\right]}^{-1}+O\left(\frac{1}{\alpha^{2}}\right).$$

Further binomial expansion leads to

(52) $${u_{1}|}_{r,\theta \to 0}=6\left\{1-\frac{5}{\alpha \phi }\left(\phi -\frac{4}{5}\right)\left(\phi +\frac{3}{2}\right)\right\}+O\left(\frac{1}{\alpha^{2}}\right).$$

This indicates a theoretical maximum of $6U_{\infty }$ for the liquid region velocity, assuming that $\phi >\frac{4}{5}$. It is of course understood that the value $\phi >\frac{4}{5}$ is based on the asymptotic expansion in 1/α up to O(1/α).

##### Porous Region Minimum Velocity Location

In this section, the radial distance at which the minimum occurs is derived asymptotically. In the Brinkman–Stokes model, we set $\partial v_{2}/\partial r=0$ which leads to:

(53) $$\begin{array}{l} \;-\left(\frac{\phi }{2\left(\phi +\frac{3}{2}\right)}+\frac{\phi }{2\left(\phi +\frac{3}{2}+1\right)}\frac{1}{\alpha }+\frac{3}{\alpha^{2}}+\frac{3}{\alpha^{3}}\right)+e^{-\alpha \left(r-1\right)}\left(r^{3}+\frac{2r^{2}}{\alpha }+\frac{3r}{\alpha^{2}}+\frac{3}{\alpha^{3}}\right)=0.\\ \; \end{array}$$

As one can observe in Fig. 6, for a large α, velocity reaches its minimum right near the interface (r=1).

(54) $$\begin{array}{ll} \text{To}\;\text{solve}\;\text{Equation}\;(53)\;\text{we}\;\text{set:} & r=1+r_{1}\epsilon +r_{2}\epsilon^{2}+\ldots \;.\\ \text{Defining}\;\epsilon =\frac{1}{\alpha }\ll 1, & r=1+r_{1}\frac{1}{\alpha }+r_{2}\frac{1}{\alpha^{2}}+\ldots \;. \end{array}$$

We substitute (54) into Eq. (53) and obtain the following

(55) $$\;-\left(\frac{\phi }{2\left(\phi +\frac{3}{2}\right)}+\frac{\phi }{2\left(\phi +\frac{3}{2}+1\right)}\frac{1}{\alpha }+\frac{3}{\alpha^{2}}+\frac{3}{\alpha^{3}}\right)+e^{-r_{1}}e^{-\frac{r_{2}}{\alpha }}e^{-\frac{r_{3}}{\alpha^{2}}}[(1+\frac{r_{1}}{\alpha }{+\frac{r_{2}}{\alpha^{2}})}^{3}+\frac{2}{\alpha }{\left(1+\frac{r_{1}}{\alpha }+\frac{r_{2}}{\alpha^{2}}\right)}^{2}+\frac{3}{\alpha^{2}}+\frac{3}{\alpha^{3}}]=0.$$

All exponential terms in Eq. (55) except $e^{-r_{1}}$ are expanded using

$$e^{-\epsilon }=1-\epsilon +\frac{\epsilon^{2}}{2!}-\ldots .$$

(56) $$\begin{array}{ll} \text{For}\;\text{the}\;\text{leading}\;\text{order},\;\text{we}\;\text{obtain:} & \frac{\phi }{2\left(\phi +\frac{3}{2}\right)}=e^{-r_{1}},\\ \text{resulting}\;\text{in} & r=1+\frac{1}{\alpha }\text{ln}\left(2+\frac{3}{\phi }\right)+O\left(\frac{1}{\alpha^{2}}\right). \end{array}$$

This clearly indicates that the minimum velocity on the equatorial plane in the porous region occurs just outside the interface. For the parameters in this study, $r-1=\left(1.12-1.56\right)\times 10^{-6}$ for 0.8≤ϕ≤2.0.

#### 4.3.2 Velocity Distribution at Various Latitudes

**Fig. 7:**
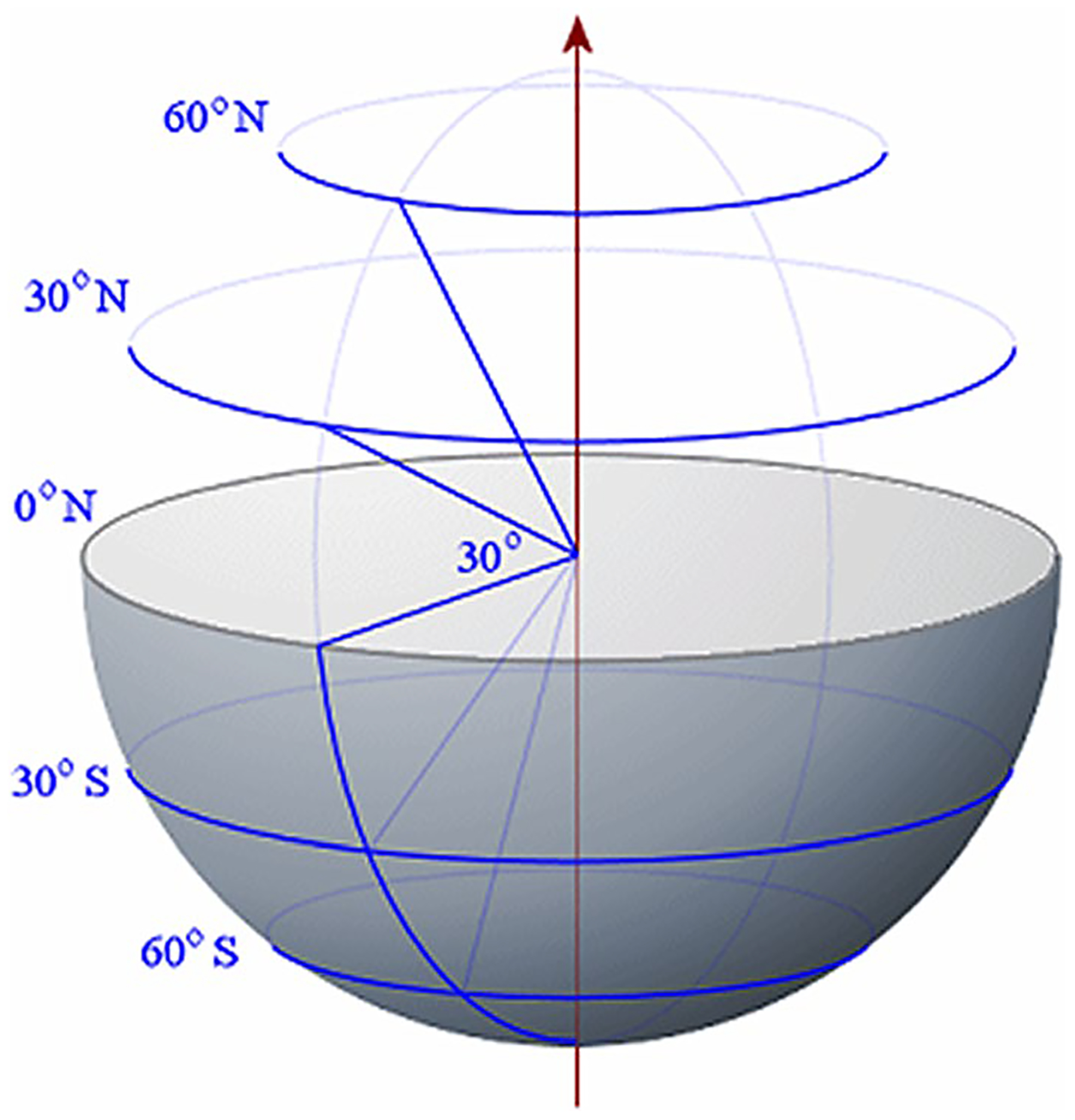
Latitude planes corresponding to $\theta^{\prime }=30{}^{\circ}$ and $\theta^{\prime }=60{}^{\circ}$. Note that $\theta^{\prime }=\frac{\pi }{2}-\theta$

**Fig. 8:**
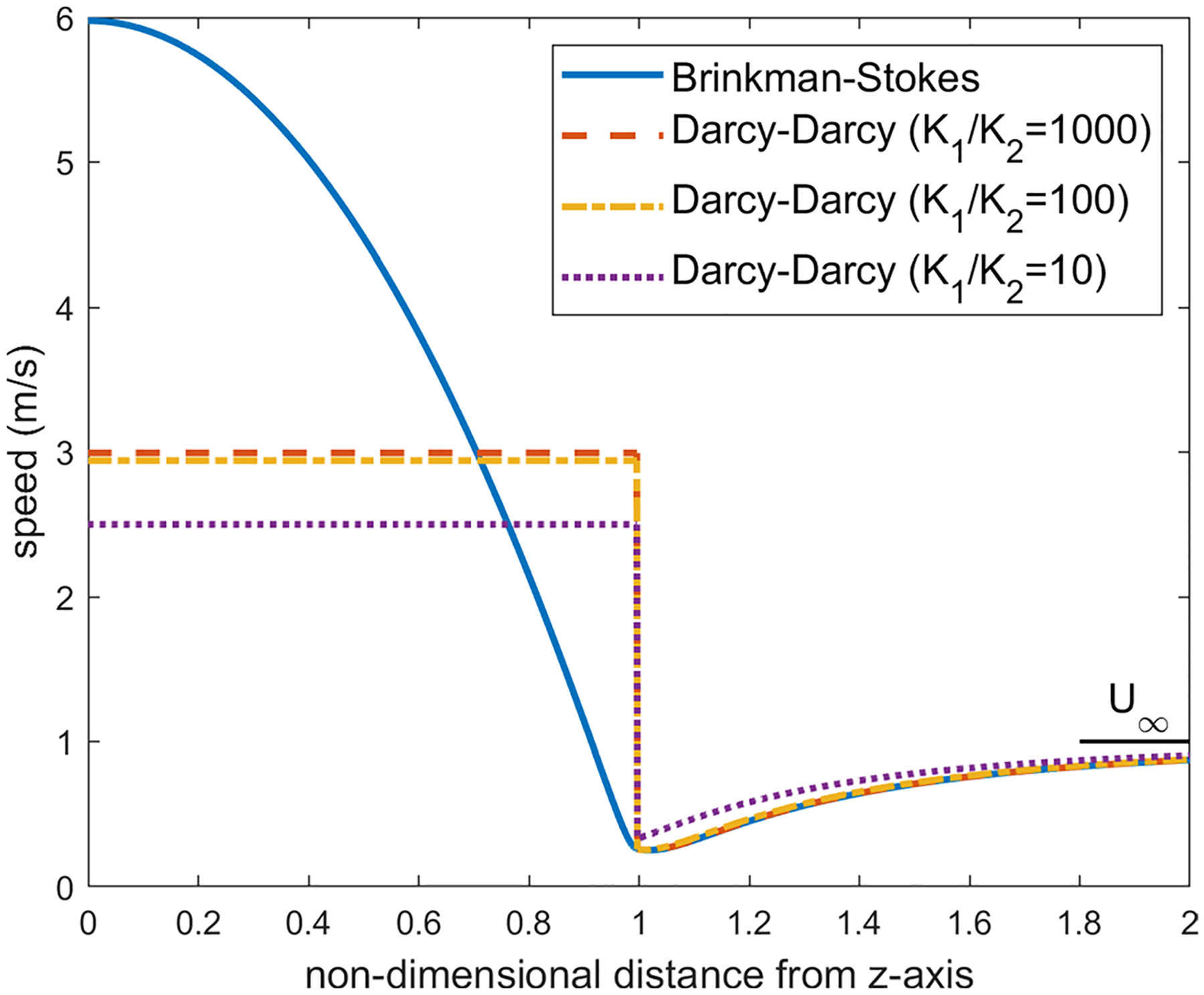
Distribution of the velocity magnitude in the latitude plane at $\theta^{\prime }=5{}^{\circ}$ (see Fig. 7) for ϕ=1.2, a=1cm and $\alpha =1.12\times 10^{6}$

**Fig. 9:**
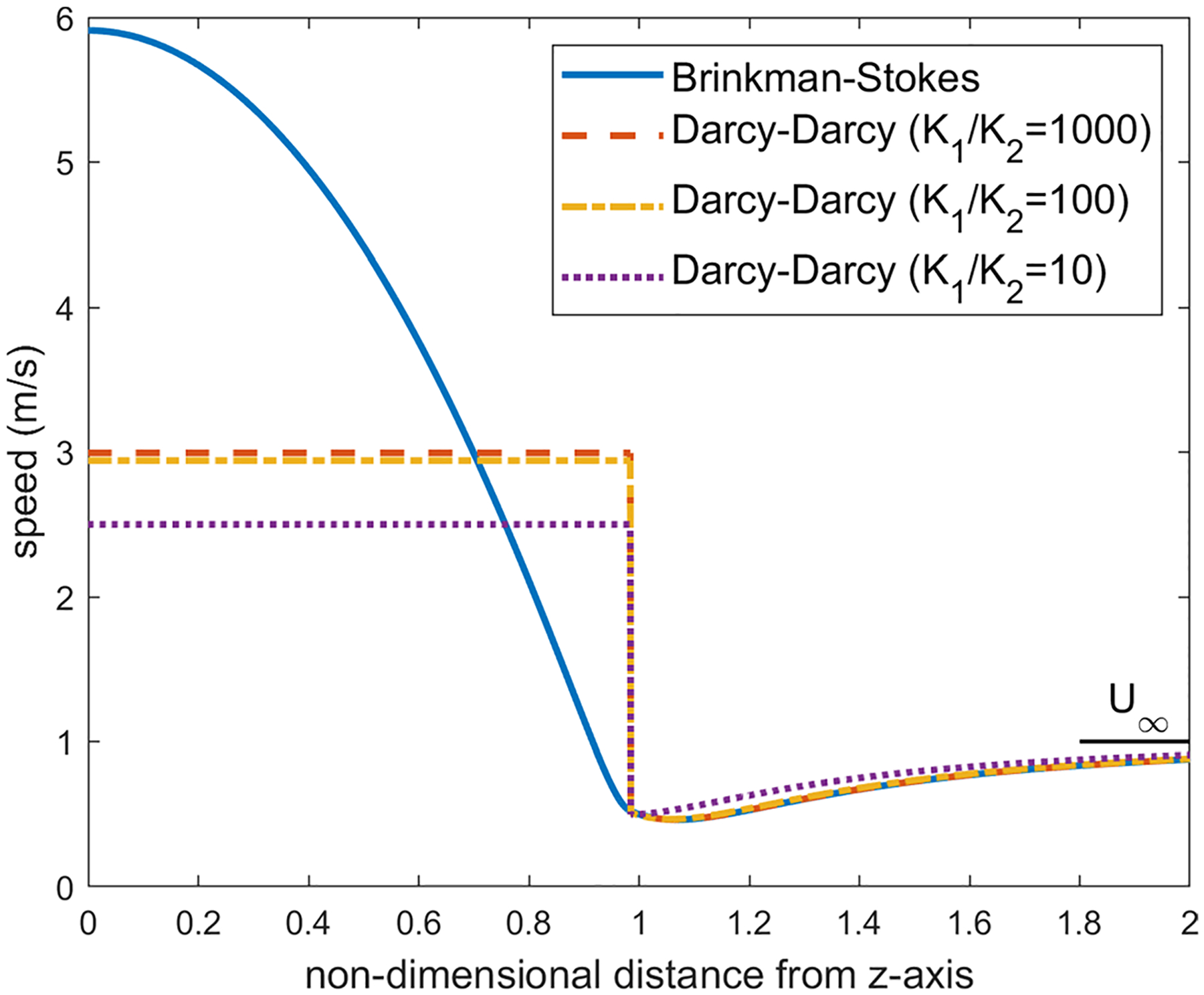
Distribution of the velocity magnitude in the latitude plane at $\theta^{\prime }=10{}^{\circ}$ (see Fig. 7) for ϕ=1.2, a=1cm and $\alpha =1.12\times 10^{6}$

**Fig. 10:**
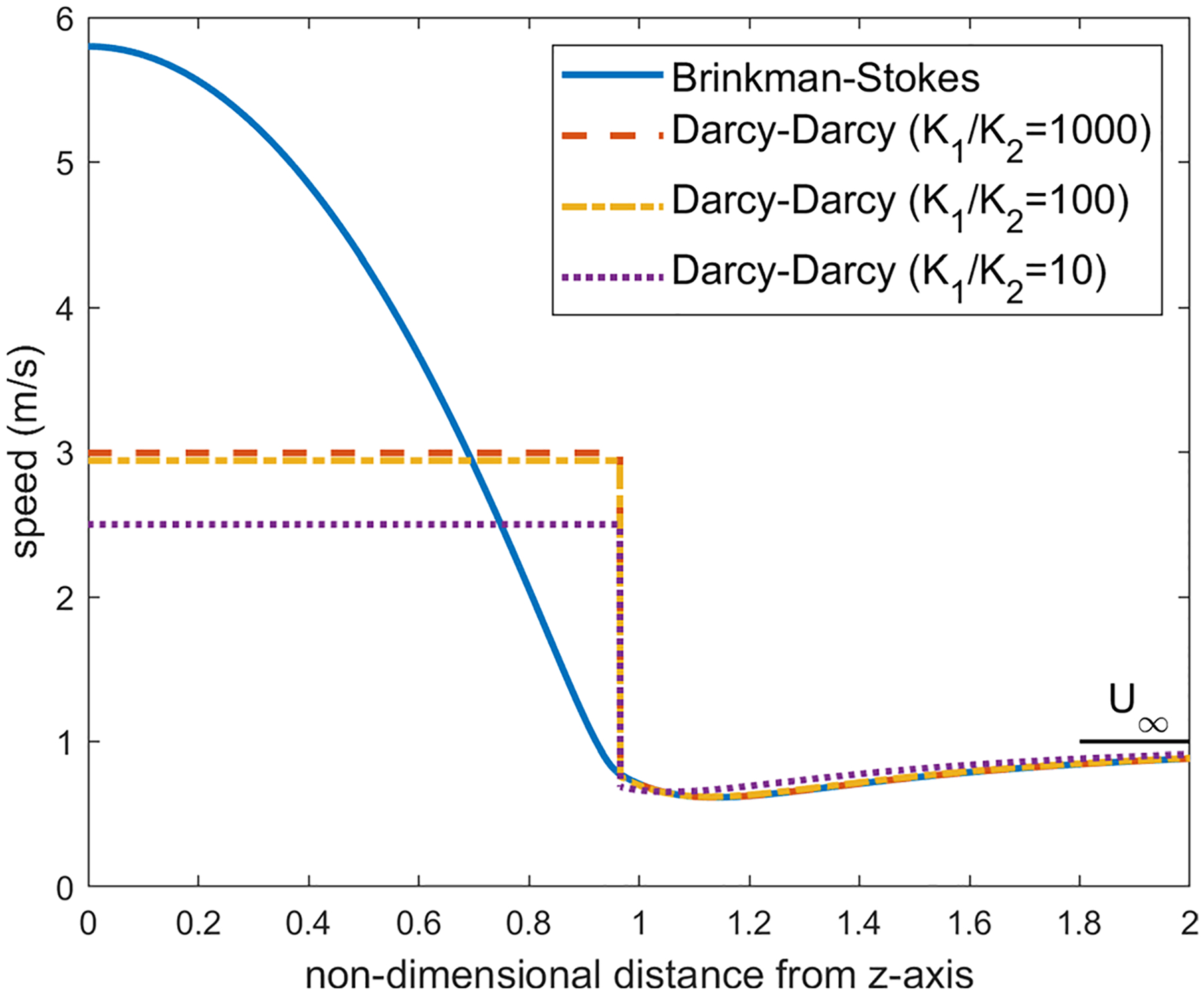
Distribution of the velocity magnitude in the latitude plane at $\theta^{\prime }=15{}^{\circ}$ (see Fig. 7) for ϕ=1.2, a=1cm and $\alpha =1.12\times 10^{6}$

**Fig. 11:**
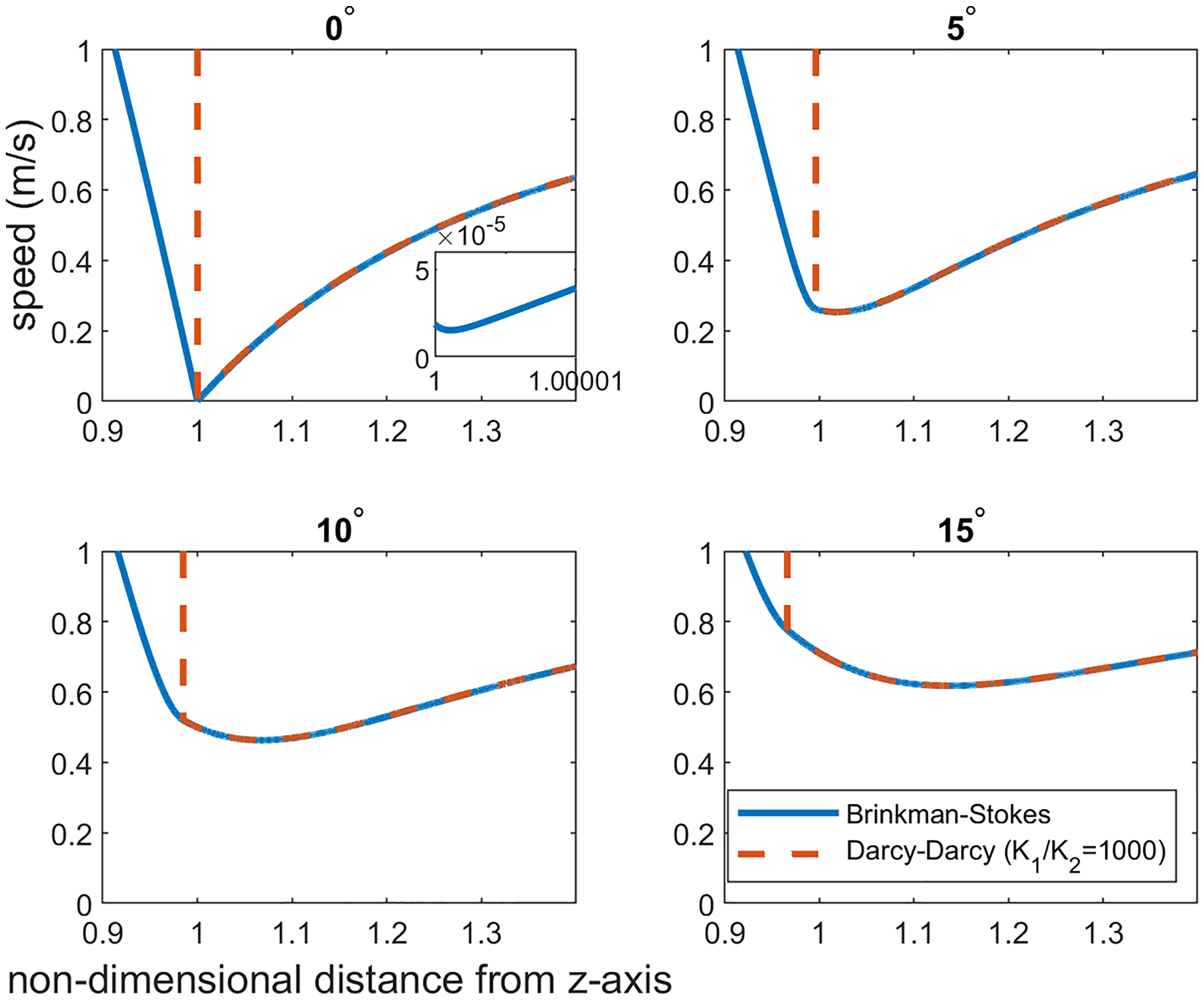
The exterior region velocity distribution in the vicinity of the interface (1≤r≤1.4) is exhibited in detail for $\theta^{\prime }=0{}^{\circ},5{}^{\circ},10{}^{\circ},15{}^{\circ}$. The location at which the minimum velocity occurs drifts further away into the porous region, from the interface, at higher latitude planes. For $\theta^{\prime }=0{}^{\circ}$, the plot is the zoomed in on the interface, at the porous side, where the velocity reaches its minimum ($r=1+1.34\times 10^{-6}$ for ϕ=1.2)

The velocity distribution over different latitude planes (see Fig. 7) has also been obtained. Once again, the calculations were carried out for a=1.0cm,$\alpha =1.12\times 10^{6}$ and ϕ in the range 0.8≤ϕ≤2.0. Here, since the velocity direction varies radially, we have calculated the velocity magnitude. The velocity distribution varies little with ϕ in this range and we are reporting the calculations for ϕ=1.2. As we move up on the latitude planes ($\theta^{\prime }=5{}^{\circ},10^{{}^{\circ}}$ and 15° in Figs. 8, 9 and 10), this minimum location moves further away from the interface toward the porous region. This can be seen more clearly in Fig. 11 where the detail in the region 1<r<1.4 is shown for the Brinkman–Stokes model. The vertical broken lines near r=1 are actually the Darcy–Darcy model results where there is a velocity discontinuity (slip) at the interface. The plot for the equatorial plane $\left(\theta^{\prime }=0\right)$ appears to be monotonic. However, more detailed calculations reveal that it does indeed have a minimum point consistent with Eq. (56) as shown in the inset in the range of 1≤r≤1.00001. For this set of plots, there is some variation with ϕ as to the location of the minimum point. This is to be expected as seen in Equation (56). Remarkably, the Darcy–Darcy exterior velocity profile falls almost exactly on the Brinkman–Stokes field for $K_{1}/K_{2}>100$. As noted before, the flow prefers to move through the path of least resistance and the streamlines tend to converge into the liquid sphere. Due to this phenomenon, in the porous region near the interface the streamlines are slightly less dense which is also manifested by speed dropping around this location.

**Fig. 12:**
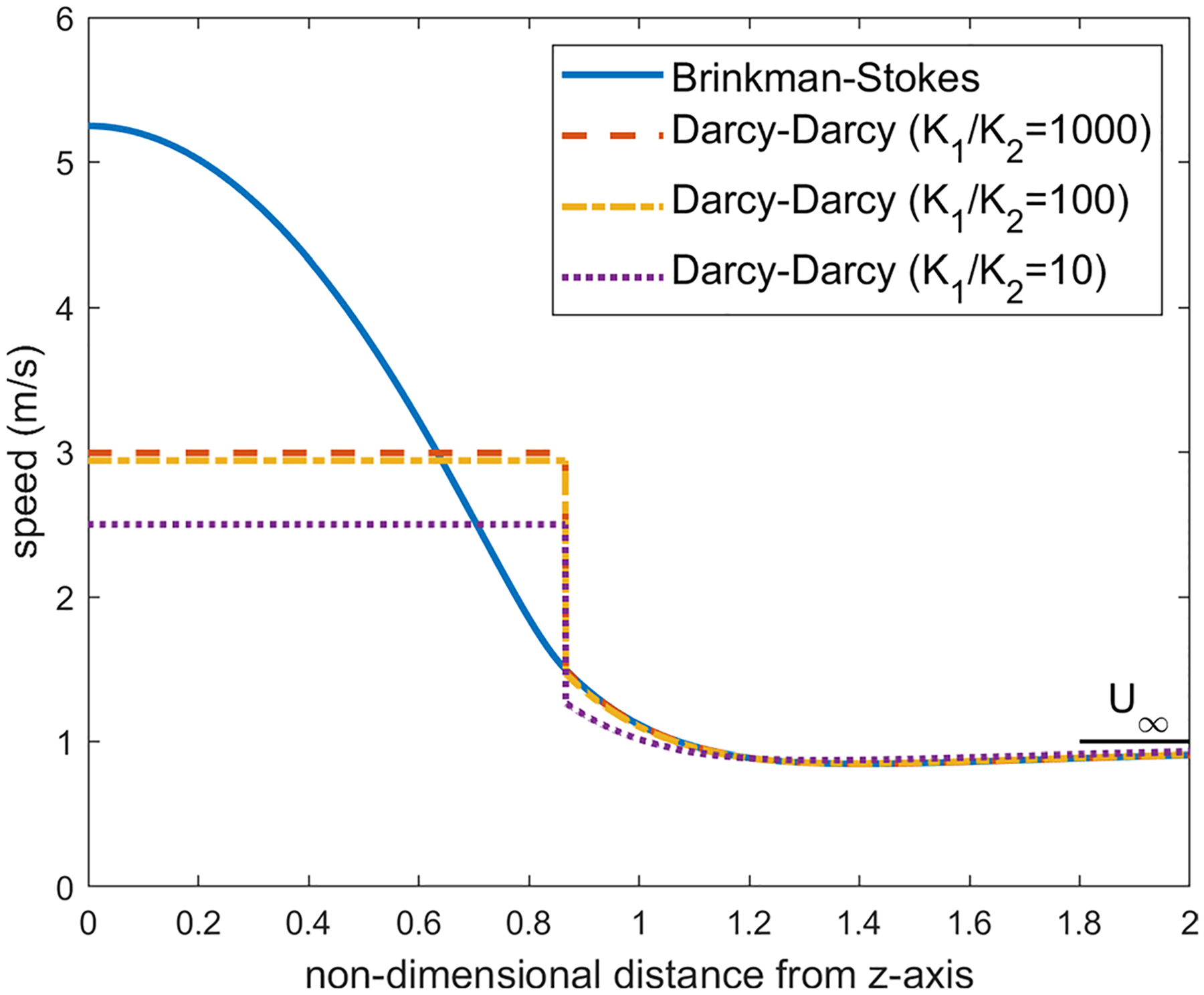
Distribution of the velocity magnitude in the latitude plane at θ=30° (see Fig. 7) for ϕ=1.2, a=1cm and $\alpha =1.12\times 10^{6}$

**Fig. 13:**
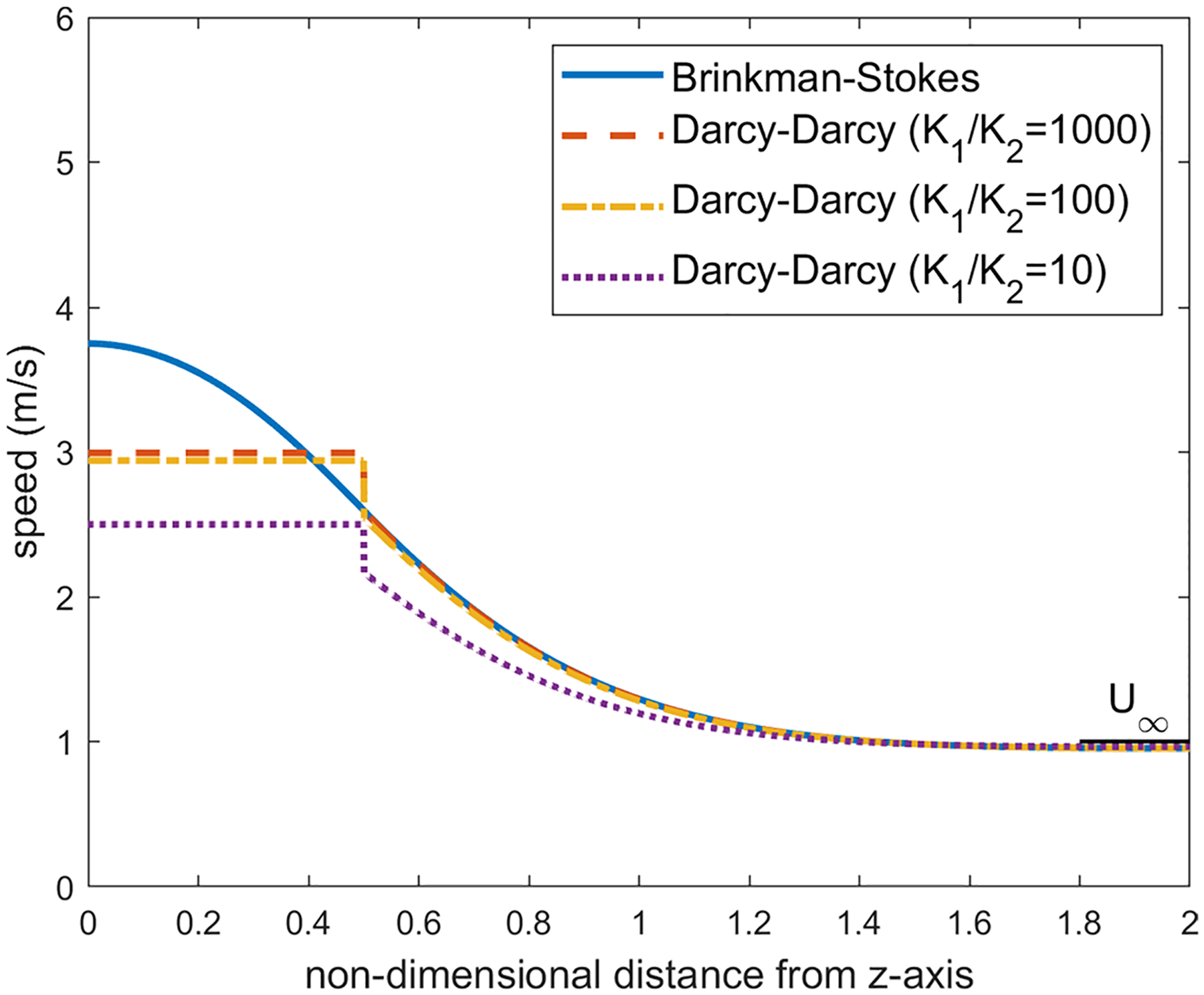
Distribution of the velocity magnitude in the latitude plane at θ=60° (see Fig. 7) for ϕ=1.2, a=1cm and $\alpha =1.12\times 10^{6}$

The results at the higher latitude planes ($\theta^{\prime }=30{}^{\circ}$ and 60°) are given in Figs. 12 and 13. At these latitude planes, for both models, the speed decreases with the horizontal distance from the interface, reaches a minimum and then increases to the far-field value at r→∞. As with the lower latitudes (Figs. 8–10), the minimum points move further out with increase in latitudes.

## 5 Conclusion

A fully analytical solution for flow in an infinite porous medium with a spherical liquid inclusion has been obtained. The porous region was modeled as Brinkman flow and spherical inclusion as Stokes flow. In addition, a simpler Darcy flow in both regions with a very high permeability in the spherical inclusion was examined and the two types of flow were compared. There are some similarities in the flow characteristics, especially in the porous region. In fact, in the porous region with $K_{1}/K_{2}\geq 100$, the flow field can be approximated very well with Darcy–Darcy model.

As expected in the Darcy–Darcy model, the liquid region flow is constant velocity while the Brinkman–Stokes has a distinct paraboloidal profile. However for modeling purposes, we have established that the total flow across the liquid sphere is equal in the two cases if the Darcy–Darcy model is taken as porous with thousands times the permeability of the porous region. However, for modeling parameters such as drug transport in the vitreous, if the liquid region velocity is required in detail, the Brinkman–Stokes model is recommended.

The fully analytical solution for the Brinkman–Stokes model has facilitated the asymptotic expansions of the velocity field for large values of the parameter α and brought out several interesting characteristics. With the liquid inclusion, the lower flow resistance permits the fluid to converge into it at the cost of lowering the flow velocity in the immediate vicinity of the liquid sphere. Of course, further away from the sphere the flow velocity catches up to the far-field. This results in a local minimum in the velocity outside the sphere. At the equatorial plane, the minimum is just by the interface. Within the sphere, the peak is of course at the axis and for the parameters we used, this is approximately six times the far-field velocity. In fact, the peak velocity of 6 $U_{\infty }$ is the limit as α→∞.

While the current results represent an axisymmetric three-dimensional model for the flow field, it is possible to extend the analysis to fully asymmetric flow using vector-potential formulation (see Sadhal (1993)).

## A Appendix

### A.1 Brinkman–Stokes Model

The derivation of the pressure and streamfunction for the porous region and spherical region is presented here.

#### A.1.1 Interior Liquid Region

In this subsection, we solve Eqs. (11), (12) and (13) in Sect. 2.1.2.

Equation (13) is a standard Stokes flow problem with the solution:

(57)
$$\psi_{1}=\left(Ar^{4}+Br^{2}\right)\;{\text{sin}}^{2}\;\theta ,$$

where, with the far-field boundary condition (3), the interior flow follows a similar behavior in θ.

The velocities may be expressed as:

(58)
$$u_{1}=2\left(Ar^{2}+B\right)\text{cos}\;\theta ,$$

(59)
$$v_{1}\;=\left(-4Ar^{2}-2B\right)\text{sin}\;\theta .$$

#### A.1.2 Exterior Porous Region

In this section of the Appendix, the derivations for pressure and streamfunction in the porous region of the Brinkman–Stokes model are shown.

Based on the study conducted by Grosan et al. (2010), the streamfunction in Eq. (17) may be expressed as:

(60)
$$\psi_{2}=\left[\frac{1}{2}r^{2}+\frac{C}{r}+Ee^{-\alpha r}\left(\frac{1}{\alpha }+\frac{1}{\alpha^{2}r}\right)\right]{\text{sin}}^{2}\;\theta ,$$

where we have chosen to write the spherical Bessel function $k_{0}\left(\alpha r\right)$ in the standard exponential form.

The velocity components may be expressed as:

(61)
$$u_{2}=\frac{1}{r^{2}\;\text{sin}\;\theta }\frac{\partial \psi_{2}}{\partial \theta }=\left[1+\frac{2C}{r^{3}}+\frac{2E}{r^{2}}e^{-\alpha r}\left(\frac{1}{\alpha }+\frac{1}{\alpha^{2}r}\right)\right]\text{cos}\;\theta ,$$

(62)
$$v_{2}=-\frac{1}{r\;\text{sin}\;\theta }\frac{\partial \psi_{2}}{\partial r}=\left[-1+\frac{C}{r^{3}}+Ee^{-\alpha r}\left(\frac{1}{r}+\frac{1}{\alpha r^{2}}+\frac{1}{\alpha^{2}r^{3}}\right)\right]\text{sin}\;\theta .$$

This leads to the following expression for the shear stress in zone 2:

(63)
$$\tau_{r\theta 2}=\frac{1}{r}\frac{\partial u_{2}}{\partial \theta }+\frac{\partial v_{2}}{\partial r}-\frac{v_{2}}{r}\;=\left[\frac{-6C}{r^{4}}-Ee^{-\alpha r}\left(\frac{\alpha }{r}+\frac{3}{r^{2}}+\frac{6}{\alpha r^{3}}+\frac{6}{\alpha^{2}r^{4}}\right)\right]\text{sin}\;\theta .$$

Using the velocity expressions (61) and (62) in Eq. (16) and integrating with respect to θ, we obtain the pressure as:

(64)
$$P_{2}=\left[-\frac{\alpha^{2}r}{\phi }+\frac{C\alpha^{2}}{\phi r^{2}}+Ee^{-\alpha r}\left(\frac{1}{\phi }-1\right)\left(\alpha^{2}+\frac{\alpha }{r}+\frac{1}{r^{2}}\right)\right]\text{cos}\;\theta ,$$

where $\phi =\tilde{\mu }/\mu$. Now, the normal stress $\tau_{rr2}$ can be expressed as:

(65)
$$\tau_{rr2}=-P_{2}+2\frac{\partial u_{2}}{\partial r}=\{\frac{\alpha^{2}r}{\phi }+C\left(\frac{-\alpha^{2}}{\phi r^{2}}-\frac{12}{r^{4}}\right)+Ee^{-\alpha r}[\alpha^{2}\left(1-\frac{1}{\phi }\right)+\frac{\alpha }{r}\left(1-\frac{1}{\phi }\right)-\frac{1}{r^{2}}\left(3+\frac{1}{\phi }\right)-\frac{12}{\alpha r^{3}}]\}\text{cos}\;\theta .$$

For the fully liquid interior, the corresponding expressions are:

(66)
$$\tau_{r\theta 1}=-6Ar\;\text{sin}\;\theta ,$$

(67)
$$P_{1}=20Ar\;\text{cos}\;\theta ,$$

(68)
$$\tau_{rr1}=-12Ar\;\text{cos}\;\theta .$$

#### A.1.3 Interface Conditions

We now go on to satisfy the boundary and interface conditions (4)–(8).

Matching the normal velocities (Eq. 5) leads to:

(69)
$$A+B-C-Ee^{-\alpha }\left(\frac{1}{\alpha }+\frac{1}{\alpha^{2}}\right)=\frac{1}{2}.$$

The continuity of tangential velocities (Eq. 6) gives:

(70)
$$4A+2B+C+Ee^{-\alpha }\left(1+\frac{1}{\alpha }+\frac{1}{\alpha^{2}}\right)=1.$$

With the shear stress continuity (Eq. 7), we have:

(71)
$$\frac{A}{\phi }=C+Ee^{-\alpha }\left(\frac{\alpha }{6}+\frac{1}{2}+\frac{1}{\alpha }+\frac{1}{\alpha^{2}}\right),$$

and normal stress continuity (Eq. 8) leads to:

(72)
$$\begin{array}{c} \;-A+C\left(\frac{\alpha^{2}}{12}+\phi \right)+Ee^{-\alpha }[\frac{\alpha^{2}}{12}\left(1-\phi \right)+\frac{\alpha }{12}\left(1-\phi \right)+\frac{1}{12}\left(1+3\phi \right)+\frac{\phi }{\alpha }+\frac{\phi }{\alpha^{2}}]=\frac{\alpha^{2}}{12} \end{array}.$$

Solving the system of four Eqs. (69)–(72), we obtain the following expressions for A,B,C and E:

(73)
$$E=\frac{-\frac{e^{\alpha }}{\alpha }}{\left(\frac{\phi }{6\left(\phi +\frac{3}{2}\right)}+\frac{3\phi }{\alpha^{2}}\right)\left(1+\frac{1}{\alpha }\right)+\left(\phi -\frac{2}{3}\right)\frac{1}{\alpha }},$$

(74)
$$C=\frac{\left(\frac{1}{6}\frac{\phi }{\phi +\frac{3}{2}}+\frac{1}{\alpha^{2}}\right)\left(1+\frac{1}{\alpha }\right)+\frac{1}{3\alpha }}{\left(\frac{\phi }{6\left(\phi +\frac{3}{2}\right)}+\frac{3\phi }{\alpha^{2}}\right)\left(1+\frac{1}{\alpha }\right)+\left(\phi -\frac{2}{3}\right)\frac{1}{\alpha }},$$

(75)
$$A=\frac{-\frac{1}{4}\frac{\phi }{\phi +\frac{3}{2}}\left(1+\frac{1}{\alpha }\right)}{\left(\frac{\phi }{6\left(\phi +\frac{3}{2}\right)}+\frac{3\phi }{\alpha^{2}}\right)\left(1+\frac{1}{\alpha }\right)+\left(\phi -\frac{2}{3}\right)\frac{1}{\alpha }},$$

and,

(76)
$$B=\frac{1}{2}+\frac{\frac{5}{12}\frac{\phi }{\phi +\frac{3}{2}}\left(1+\frac{1}{\alpha }\right)+\frac{1}{3\alpha }}{\left(\frac{\phi }{6\left(\phi +\frac{3}{2}\right)}+\frac{3\phi }{\alpha^{2}}\right)\left(1+\frac{1}{\alpha }\right)+\left(\phi -\frac{2}{3}\right)\frac{1}{\alpha }}.$$

The use of these coefficients in Eqs. (57), (60), (64) and (67) results in the explicit expressions for pressure and streamfunction in the both regions which are presented in Sect. 2.2.

### A.2 Darcy–Darcy Model

In this section, we have gone in depth of the derivations for the pressure and velocity in the Darcy–Darcy model.

The governing Eq. (22) in Sect. 3 is written in spherical coordinates and dimensionless form (using the dimensionless parameters in Eq. (1) so that

(77)
$$\frac{\partial^{2}P_{i}}{\partial r^{2}}+\frac{2}{r}\frac{\partial P_{i}}{\partial r}+\frac{1}{r^{2}}\frac{\partial }{\partial \bar{\mu }}\left[\left(1-{\bar{\mu }}^{2}\right)\frac{\partial P_{i}}{\partial \bar{\mu }}\right]=0,\;i=1,2,$$

where $\bar{\mu }=\text{cos}\;\theta$.

In terms of Legendre polynomials, pressure in liquefied and porous regions is expressed as:

(78)
$$P_{1}=\overset{\infty }{\underset{n=0}{\sum }}C_{n}\;r^{n}{\text{P}}_{n}\left(\bar{\mu }\right),$$

(79)
$$\begin{array}{c} P_{2}=\overset{\infty }{\underset{n=0}{\sum }}\left(A_{n}\;r^{n}+\frac{B_{n}}{r^{n+1}}\right){\text{P}}_{n}\left(\bar{\mu }\right). \end{array}$$

Using the far-field condition (Eq. 25),

(80)
$$P_{2}=\frac{-a^{2}}{K_{2}}r\bar{\mu }+\overset{\infty }{\underset{n=0}{\sum }}\frac{B_{n}}{r^{n+1}}{\text{P}}_{n}\left(\bar{\mu }\right).$$

To derive the $B_{n}$ and $C_{n}$ values, the interface conditions at r=1 are satisfied (Eqs. (78) and (80)).

The final expression for pressure is presented in Sect. 3.

## List of Symbols

${\bar{P}}_{i}$: Pressure
${\bar{\text{V}}}_{i}$: Velocity vector
$P_{i}$: Non-dimensional pressure
${\text{V}}_{i}$: Non-dimensional velocity vector
μ: Dynamic viscosity
$\tilde{\mu }$: Brinkman viscosity
ϕ: Ratio of Brinkman viscosity to dynamic viscosity
$K_{1}$: Darcy coefficient of the liquid region in the Darcy–Darcy model
$K_{2}$: Darcy coefficient of the porous region
a: Radius of the spherical liquid region
$U_{\infty }$: Far-field velocity
$u_{i}$: Non-dimensional radial velocity
$v_{i}$: Non-dimensional tangential velocity
$\psi_{i}$: Non-dimensional streamfunction
${\bar{\tau }}_{r\theta i}$: Shear stress
${\bar{\tau }}_{rri}$: Normal stress
$\tau_{r\theta i}$: Non-dimensional shear stress
$\tau_{rri}$: Non-dimensional normal stress
α: $\frac{a}{\sqrt{K_{2}}}$
$\bar{r}$: Radial coordinate
r: Non-dimensional radial coordinate
θ: Polar angle
$\theta^{\prime }$: Latitude angle
$\bar{\mu }$: cosθ
${\text{P}}_{n}\left(\bar{\mu }\right)$: Legendre polynomial of the order n
$\bar{Q}$: Volumetric flow across the liquid sphere

## Subscripts

i=1: Liquid region
i=2: Porous region
